# Optimization of Fermentation Conditions and Properties of an Exopolysaccharide from *Klebsiella* sp. H-207 and Application in Adsorption of Hexavalent Chromium

**DOI:** 10.1371/journal.pone.0053542

**Published:** 2013-01-08

**Authors:** Li Qiang, Li Yumei, Han Sheng, Liu Yingzi, Song Dongxue, Hao Dake, Wang Jiajia, Qu Yanhong, Zheng Yuxia

**Affiliations:** 1 School of Medicine and Life Sciences, University of Jinan, Jinan, China; 2 School of Chemistry and Chemical Engineering, University of Jinan, Jinan, China; 3 Department of Criminal science and Technology, Shandong Police College, Jinan, China; Montana State University, United States of America

## Abstract

The novel exopolysaccharide HZ-7 is produced by *Klebsiella* sp. H-207, and its fermentation conditions were optimized by response surface methodology (RSM). In this study, the optimized medium consisted of sucrose 31.93 g/L, KNO_3_ 2.17 g/L and K_2_HPO_4_ 5.47 g/L; while the optimized culture conditions consisted of seed age 13 h, with an inoculum size of 10.6% and incubation temperature of 28.9°C. A maximum HZ-7 yield of about 15.05 g/L was achieved under the optimized conditions using RSM and single-factor experiments. Next the exopolysaccharide HZ-7 was partially purified and characterized. The resulting product showed good properties, such as high concentration of uronic acid (41.67%), low average molecular weight (about 1.94×10^5 ^Da) and porous surface structure, were very advantageous to biosorption. Therefore HZ-7 was applied to absorb hexavalent chromium (Cr(VI)). The maximum adsorption efficiency (99.2%) which was obtained at an initial pH of 1.0 along with an initial Cr(VI) concentration of 20 mg/L, was not affected by ordinary metal ions and temperature. These data suggest *Klebsiella* sp. H-207 exopolysaccharide will be promising potential for industrial application.

## Introduction

Microbial exopolysaccharides (EPSs) are principally composed of carbohydrate polymers, and they are produced by many microorganisms including bacteria, fungi and yeasts [1]–[3]. They have been extensively applied in various biotechnological fields such as pharmaceutical, cosmetics, food, textile, oil recovery, metal mining and metal recovery [4]. These EPSs have many predominant advantages, such as easy production, better chemical and physical properties, cost effectiveness and supply. Regardless, only few EPSs have achieved great commercial success, either because of their high production costs and low yields or due to difficulties in finding new practical applications [5].

Several studies have reported that the yield and quality of microbial EPSs are greatly affected by the nutritional and environmental conditions. These studies also suggest that an increase in EPSs production is possible by manipulating the culture conditions [6]–[8]. To achieve this aim, the response surface methodology (RSM), as a powerful statistical tool, has been widely selected to improve product yields, reduce overall process time, and costs. Compared with conventional methods, RSM can be used to design experiments, build models, search optimum factors for desirable responses, and evaluate the relative significance of several influence factors even in the presence of complex interactions [9].

Recently, the application of EPSs was expanded to the removal of toxic heavy metal pollutants [10]–[12], especially for the removal of hexavalent chromium (Cr(VI)). Cr(VI) exists in the form of Cr_2_O_7_^−^, which is widely present in wastewater from several industries such as pigment and dye production, leather tanning, electro-plating, wood treatment, textile dyeing, and the steel industry. In this form, Cr(VI) is highly toxic to living organisms and potentially carcinogenic to humans [13]. However, traditional treatment methods were insensitive to less than 100 mg/L of Cr(VI). In contrast, biosorption of microbial EPSs is fit to outcome this drawback [10]–[12].

In present study, a novel exopolysaccharide, named HZ-7, was produced by *Klebsiella* sp. H-207 that was isolated from activated sludge. A series of experiments were performed in order to investigate the HZ-7 production and its properties. Finally, HZ-7 was applied to the adsorption of Cr(VI). The conclusions are as follows: (1) the optimal medium for HZ-7 production was very simple and cost-effective; (2) the average molecular weight of HZ-7 (1.94×10^5 ^Da) was lower than the reported molecular weight of other *Klebsiella* sp. Bacteria exopolysaccharides. (More than 2.0×10^6^ [Da) [13], [14]; (3) HZ-7 can be applied to effectively adsorb low concentration (<20 mg/L) of Cr(VI).

## Materials and Methods

### Microorganism

An exopolysaccharide-producing *Klebsiella* sp. H-207 was isolated from an activated sludge sample. It was identified by analyzing its physiological and biochemical characteristics as well as the 16S rDNA sequence (GenBank accession number JX455816).

### Media and Culture Conditions

The medium for agar slant consisted of (g/L) yeast extract, 5; peptone, 10; NaCl, 20; and agar, 20. The seed medium contained (g/L) sucrose, 10; KNO_3_, 4; NaCl, 0.1; KH_2_PO_4_, 2; K_2_HPO_4_, 5; (NH_4_)_2_SO_4_, 0.2 and MgSO_4_·7H_2_O, 0.2. The primary production medium (g/L) included sucrose, 20; KNO_3_, 3; NaCl, 0.1; KH_2_PO_4_, 2; K_2_HPO_4_, 5; (NH_4_)_2_SO_4_, 0.2 and MgSO_4_·7H_2_O, 0.2. The initial pH of the medium was adjusted to 7.0–7.5.

For seed preparation, a single colony of the strain H-207 was inoculated into 50 mL of seed medium in a 250-mL flask, and incubated at 32°C with shaking at 180 rpm for 12 h. Then the 10% (v/v) seed culture was transferred to 50 mL of production medium in a 250-mL flask, and the flask was incubated at 32°C with shaking at 180 rpm for 80 h.

### Quantification of the Exopolysaccharide

The fermentation broth was centrifuged at 8,000 *g* for 30 min at 4°C. The supernatant was collected and dried in the oven at 50°C. Thus the yield of exopolysaccharide HZ-7 was determined and represented as g/L dry weight. All data given below are the mean values based on triplicate experiments.

### Experimental Designs and Optimization

The effects of medium constituents and culture conditions on HZ-7 production were investigated by classical one-factor-at-a-time method [15]. Six carbon sources (glucose, sucrose, lactose, maltose, soluble starch and dextrin), six nitrogen sources (yeast extract, peptone, urea, ammonium, (NH_4_)_2_SO_4_, NH_4_Cl andKNO_3_) and two mineral salts (K_2_HPO_4_ and MgSO_4_·7H_2_O) were added to basal medium with the concentrations of the ingredients kept constant. Six culture conditions were optimized one by one, which included inoculum size, seed age, medium volume, agitation rate, initial pH and temperature.

After determining the significant factors, a three-level, three-factor Box-Behnken design (BBD) was applied to further study [16]. The center points and parameters were chosen according to single-factor experiments. Fifteen trials for three different variables included medium consisted of sucrose, KNO_3_ and K_2_HPO_4_; culture conditions which consisted of inoculum size, seed age and temperature were designed. Three levels, “high”, “middle ” and “low”, were evaluated for each factor and designated as coded level +1, coded level 0 and coded level −1, respectively. The design was developed and the significance of the model was evaluated and generated by Design-Expert software (version 8.0.1). All the experiments were performed at least three times, and the results are presented as mean values of three or more trials. The design is represented by a second-order polynomial regression model as follows:



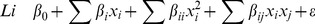
(1)where y is the predicted response, *β*_0_ the intercept, *β_i_* the linear coefficient, *β_ij_* the interactive coefficients, *β_ii_* the quadratic coefficients, and ε is the error, and *x_i_* and *x_j_* are the coded independent variables. The equation was used to generate response surface graphs for maximum production of HZ-7, and the experimental and predicted results were compared to confirm the validity of the model. Variables showing agreement between experimental and predicted results at confidence level (>90%) were considered to have a significant influence on HZ-7 production.

### Partial Purification of HZ-7

The fermentation broth was boiled for 30 min and then centrifuged at 8,000*g* for 30 min at 4°C. The supernatant was mixed with three volumes of chilled ethanol and left to stand at 4°C overnight. The resultant precipitate was collected by centrifugation at 8,000 *g* for 30 min and then washed three times using 70% (v/v) ethanol. Then the precipitate was dissolved and dialyzed using deionized water. Finally, the main polysaccharide fraction of HZ-7 (HZ-7L) was obtained using Sephacryl S-500 column chromatography (16 mm×180 mm, Amersham, US) at a flow rate of 0.2 mL/min.

### Chemical and Physical Analysis of HZ-7

The main polysaccharide fraction of HZ-7 (HZ-7L) was used for the following analysis. The total sugar content was determined by the phenol–sulphuric acid method [17], using glucose as the standard solution; uronic acids and amino sugar were determined by the carbazole–sulphate reaction and the Elson–Morgan reaction with glucuronic acid and glucose amine as the standard solution, respectively [18]. The protein content was measured by the Bradford method with bovine serum albumin as a standard [19].

Element analysis was achieved with an elemental analyzer (Perkin-Elmer; CHNS/O-2400 II). The analysis was operated with 2 mg of powdered samples at 1000°C of oven temperature for 3 min. Infrared spectra was recorded in the frequency range of 4000-400 cm^−1^ by a Fourier transform (FT-IR) spectrophotometer (Thermo Electron, Nicolet IR200). The KBr tablets were prepared in the flowing manner: the sample pellets 2 mg was mixed with 200 milligram of dry potassium bromide (KBr), then the mixture was pressed into a 16 mm diameter mold. Scanning electron microscope (SEM, LEO 1530, Germany) analysis was performed by the following method: the sample was coated with a thin layer of gold by means of ion sputter coating and then applied to scanning analysis with an accelerating voltage of 10 kV.

The average molecular weight was determined by a high performance liquid chromatography equipped with multi angle laser light scattering (Wyatt, DAWN HELEOS) and refractive index detector (Agilent, G1362A). Samples were applied to TSK gel GMPWXL column (7.8 mm×300 mm) and eluted with 0.2 mol/L NaCl at a flow rate of 0.6 mL/min, with the column temperature maintained at 35°C.

### Adsorption of Cr(VI)

The experiments for adsorption of Cr(VI) were performed in 250-mL flasks containing 20 mL of HZ-7 polysaccharide solution (10 g/L) and 30 mL of Cr(VI) solution (50 mg/L). The initial pH of the mixture was adjusted to varied pHs respectively. Then the flasks with reaction mixture were incubated at room temperature and shaken at 120 rpm for 30 min.

The effects of various parameters on the Cr(VI) adsorption were investigated by the above method. Solutions of NaCl, CaCl_2_, MgCl_2_ and AlCl_3_ were used as cation sources and the dosage along with the concentration were 5 mL and 1% (w/v), respectively. The initial pH was adjusted from 0.5 to 5. The reaction temperature was varied from 20 to 40°C, and the reaction time was varied from 10 to 80 min. The initial Cr(VI) concentration was varied from 20 to 280 mg/L. The Cr(VI) concentration was measured using the Diphenylcarbazide (DPC) method [20], using potassium dichromate as the standard solution. The adsorption efficiency was calculated according to the following equation:
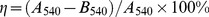
(2)where η is adsorption efficiency, A_540_ and B_540_ represent absorbance values of the initial reaction solution and the final reaction solution.

## Results and Discussion

### Kinetics of Growth and HZ-7 Production

**Figure 1:**
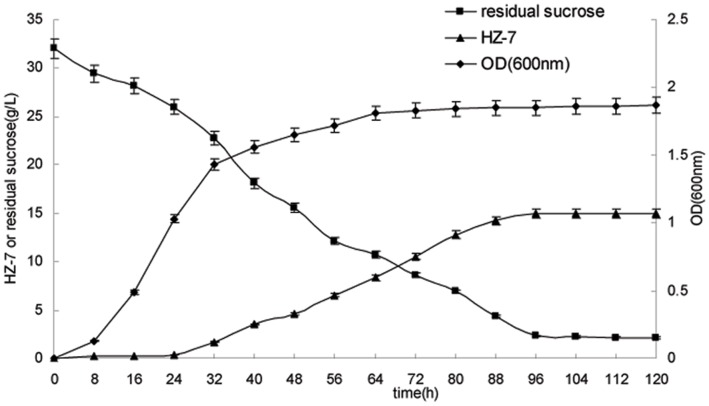
Growth profile and HZ-7 production by *Klebsiella* sp. H-207. The HZ-7 production (g/L), cell growth (OD_600_) and residual sucrose (g/L) were detected during fermentation process. The medium (g/L) consisted of sucrose, 30; KNO_3_, 3; NaCl, 0.1; KH_2_PO_4_, 2; K_2_HPO_4_, 5; (NH_4_)_2_SO_4_, 0.2 and MgSO_4_·7H_2_O, 0.2. The pH was adjusted to 7.5, the incubation temperature was 32°C, and the medium volume was 50 mL/250 mL. All the data were from triplicate experiments (mean ± SD).

The synthesis of HZ-7 as a function of the growth was investigated by monitoring the changes of cell density (OD_600_), the production of HZ-7 (g/L) and residual sucrose concentration (g/L) during the entire fermentation period. As shown in Figure 1, the residual sucrose concentration declined till close exhaustion at 96 h. The cell density rapidly reached maximum in the first 24 h, but hardly any of the HZ-7 was synthesized at this time. The HZ-7 production was gradually increased after 24 h, and its production was maintained at a constant level after 96 h. These results indicate that the synthesis of HZ-7 occurred only in the post-stationary growth phase of the strain (growth dissociated synthesis). Therefore, following optimized experiments only focused on HZ-7 production.

### Optimization of Medium for HZ-7 Production

**Figure 2:**
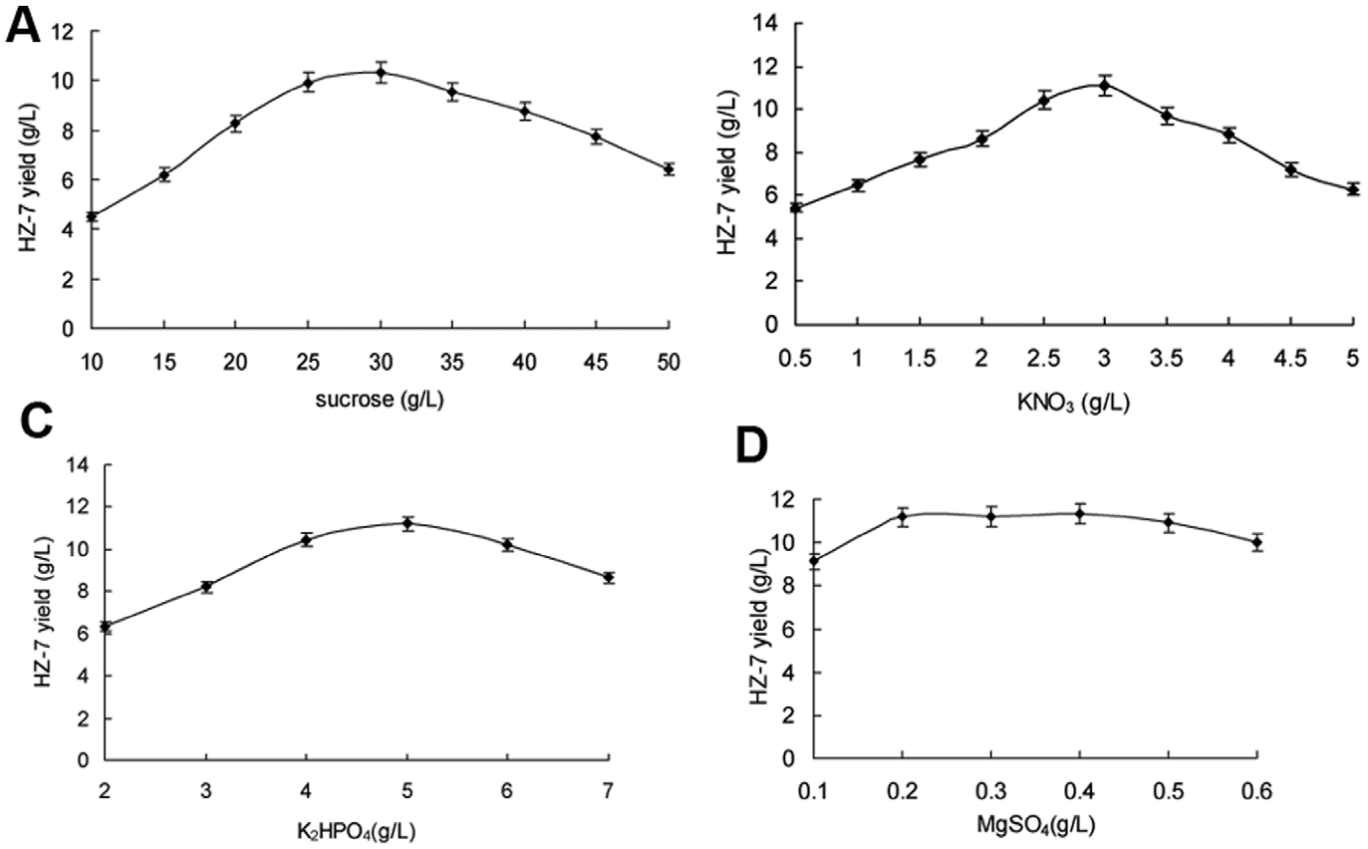
The effects of the concentration of various constituents in medium on HZ-7 production. A, sucrose (g/L); B, KNO_3_ (g/L); C, K_2_HPO_4_ (g/L); D, MgSO_4_ (g/L). Each bar represented the mean± SD of three replicates.

HZ-7 production was significantly affected by the types of carbon sources and their concentrations in the medium [21]. HZ-7 yields varied from 2.13 g/L to 9.29 g/L using different carbon sources. The highest yield (9.29 g/L) of HZ-7 was obtained when sucrose was used as the carbon source, whereas the lowest yield (2.13 g/L) occurred when dextrin was used as the sole carbon source. Glucose, soluble starch, maltose and lactose were also favorable carbon sources for HZ-7 production. Nevertheless, sucrose was most effective and economical for large scale production of HZ-7 among these carbon sources. The effect of the concentration of sucrose on HZ-7 production was further investigated (Figure 2A) and the optimal concentration of sucrose was 30 g/L with HZ-7 yield being 10.34 g/L, which is in agreement with the previous study [7].

Higher yields were achieved by the addition of inorganic nitrogen and organic nitrogen sources. Of all the nitrogen sources, KNO_3_ was found to be the most favorable for HZ-7 production and resulted in the maximum yield of HZ-7 (11.12 g/L) using 3 g/L of KNO_3_ (Figure 2B). According to previous study, low concentration of nitrogen was beneficial in the accumulation of exopolysaccharides [22]. Meanwhile, KNO_3_ as the nitrogen source was more advantageous to polysaccharide synthesis than other substances, since the potassium ion provided a stimulative effect in the glycolytic clycle [7].

The deficiency of phosphate may inhibit the glycometabolism, while the scarcity of magnesium can influence the oxidation of the carbon source [8]. Therefore, K_2_HPO_4_ and MgSO_4_ were selected as mineral salts added to the medium. The results demonstrate that HZ-7 production was improved by adding 5 g/L of K_2_HPO_4._ However both lower and higher concentration would inhibit its production (Figure 2C). HZ-7 production was maintained at a constant level when the concentration of MgSO_4_ was 0.2–0.4 g/L (Figure. 2D).

Based on above results, three key factors (sucrose, KNO_3_ and K_2_HPO_4_) significantly affected HZ-7 production, which were selected for further analysis with Box-Behnken design (BBD). The experimental designs and results of the BBD are in Table 1. The BBD was performed at least three times with various concentration ranges of each factor in order to establish confidence in the results. The following second-order ploynomial equation was established:

**Table 1:** The matrix of the BBD experiment for medium optimization and the corresponding experimental data.

Run	*X*_1_ (sucrose)		*X*_2_ (KNO_3_)		*X*_3_ (K_2_HPO_4_)		HZ-7 yeild (g/L)
	Coded level	Real level (g/L)	Coded level	Real level (g/L)	Coded level	Real level (g/L)	
1	0	30	1	3	−1	3	10.78±0.08
2	1	40	−1	1	0	5	9.85±0.06
3	0	30	1	3	1	7	12.32±0.05
4	1	40	0	2	−1	3	10.23±0.07
5	−1	20	0	2	1	7	13.53±0.04
6	1	40	0	2	1	7	10.82±0.06
7	1	40	1	3	0	5	10.68±0.07
8	0	30	−1	1	−1	3	10.21±0.06
9	−1	20	0	2	−1	3	7.25±0.08
10	−1	20	−1	1	0	5	7.64±0.07
11	0	30	−1	1	1	7	10.78±0.05
12	0	30	0	2	0	5	13.46±0.05
13	−1	20	1	3	0	5	7.89±0.02
14	−1	20	0	2	1	7	8.85±0.03
15	0	30	0	2	0	5	13.64±0.04

*X*_1_, sucrose (g/L); *X*_2_, KNO_3_ (g/L); *X*_3_, K_2_HPO_4_ (g/L).



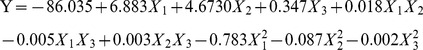
(3)where *Y* represents HZ-7 production (g/L); −86.035 is the intercept; 6.883, 4.6730 and 0.347 are the linear coefficients; 0.018, −0.005 and 0.003 are the interactive coefficients, −0.783, −0.087 and 0.002 are the quadratic coefficients; and *X*_1_, *X*_2_ and *X*_3_ are the concentrations of sucrose, KNO_3_ and K_2_HPO_4_, respectively.

**Figure 3:**
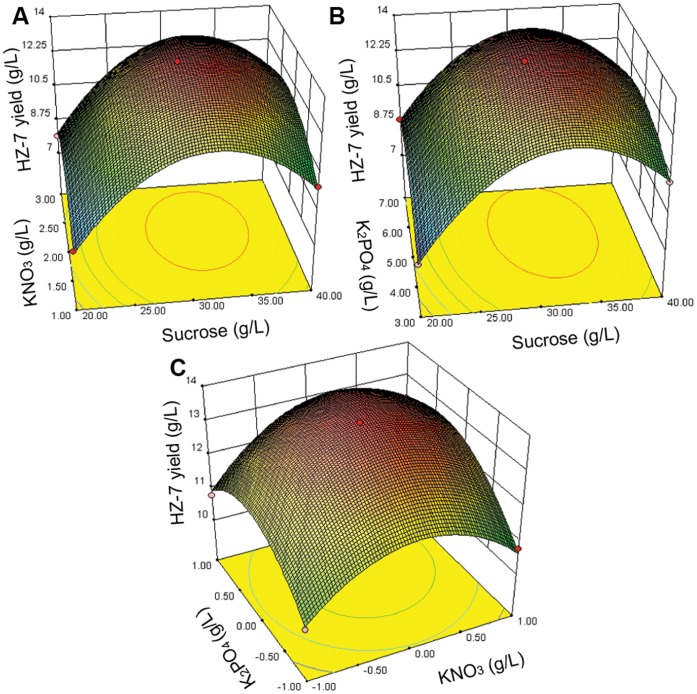
Response surface plots of the effect of three variables in medium on HZ-7 production. A, interaction of sucrose (*X*_1_, g/L) and KNO_3_ (*X*_2_, g/L); B, interaction of sucrose (*X*_1_, g/L) and K_2_HPO_4_ (*X*_3_, g/L); C, interaction of KNO_3_ (*X*_2_, g/L) and K_2_HPO_4_ (*X*_3_, g/L).

The statistical significance of equation (3) was evaluated by the *F*-test and ANOVA analysis (Table 2). The three-dimensional graphs based on Equation (3) were shown in Figure 3. The equation was very reliable with an *R* ^2^ value of 0.9975, which indicated that 99% of the variability in the response could be explained by this model. As shown in Table 2, the ‘Model *F*-value’ of 222.31 implied that the model was significant, and there was only a 0.01% chance that a ‘Model *F*-value’ could occur due to noise (*P*<0.0001). The low coefficient of variation (CV) of 1.6% also indicated that the model was reliable. Values of ‘prob>F’ less than 0.05 indicated the model terms were significant. In this case, coded values *X*_1_, *X*_2_, *X*_3_, *X*_1_^2^, *X*_2_^2^ and *X*_3_^2^ were significant model terms. The interaction of *X*_1_ *X*_2_ (sucrose/KNO_3_) was not significant (*P*>0.05), however, a substantial amount of sucrose was necessary for HZ-7 production (Figure 3A). On the other hand the interactive effect of K_2_HPO_4_/sucrose (*X*_1_ *X*_3_) and K_2_HPO_4_/KNO_3_ (*X*_2_ *X*_3_) on HZ-7 production were significant (*P*<0.05), which implied potassium and phosphate were crucial to HZ-7 synthesis (Figure 3B, C). According to the canonical analysis, the optimal concentrations of sucrose, KNO_3_ and K_2_HPO_4_ were 31.93 g/L, 2.17 g/L and 5.47 g/L, respectively. The maximum yield was estimated to be 13.54 g/L, and the actual yield obtained with the optimal medium was 13.42 g/L (the average value of triple experiments), which was in close accordance with the model prediction. In addition, the sole nitrogen source and mineral salt were used in the fermentation medium for maximum HZ-7 production, which would be advantage to reduce production cost and operation process of EPS. Also, this medium composition for EPS production was simplest among present reports which consisted of complex nitrogen sources or multiple mineral [23]–[26].

**Table 2:** Variance analysis (ANOVA) of response surface quadratic model for the medium of HZ-7 production.

Source	Sum of Squares	df	Mean Square	*F* Value	*p*-value Prob>*F*
Model	60.10	9	6.68	222.31	<0.0001^a^
*X*_1_	12.38	1	12.38	411.98	<0.0001^a^
*X*_2_	1.27	1	1.27	42.35	0.0013^a^
*X*_3_	2.31	1	2.31	76.94	0.0003^a^
*X*_1_ *X*_2_	0.084	1	0.084	2.80	0.1551
X_1_ X_3_	0.26	1	0.26	8.49	0.0333^a^
*X*_2_ *X*_3_	0.24	1	0.24	7.83	0.0381^a^
*X*_1_^2^	36.21	1	36.21	1205.52	<0.0001^a^
*X*_2_^2^	7.20	1	7.20	239.78	<0.0001^a^
*X*_3_^2^	4.67	1	4.67	155.34	<0.0001^a^
Residual	0.14	5	0.030		
Lack of Fit	0.12	3	0.045	5.05	0.1599
Pure Error	0.016	2	0.008		
Cor Total	60.23	14			

*R* ^2^ (coefficient of determination)  = 0.9975; Adj *R* ^2^ (Adjusted coefficient of determination) = 0.9934;

CV (variation coefficient) = 1.6%.

df, degree freedom.

a Model terms are significant.

### Optimization of Culture Conditions for HZ-7 Production

The effects of seed age, inoculum size, medium volume, agitation rate, initial pH, and incubation temperature on HZ-7 production were investigated by single-factor experiments.

**Figure 4:**
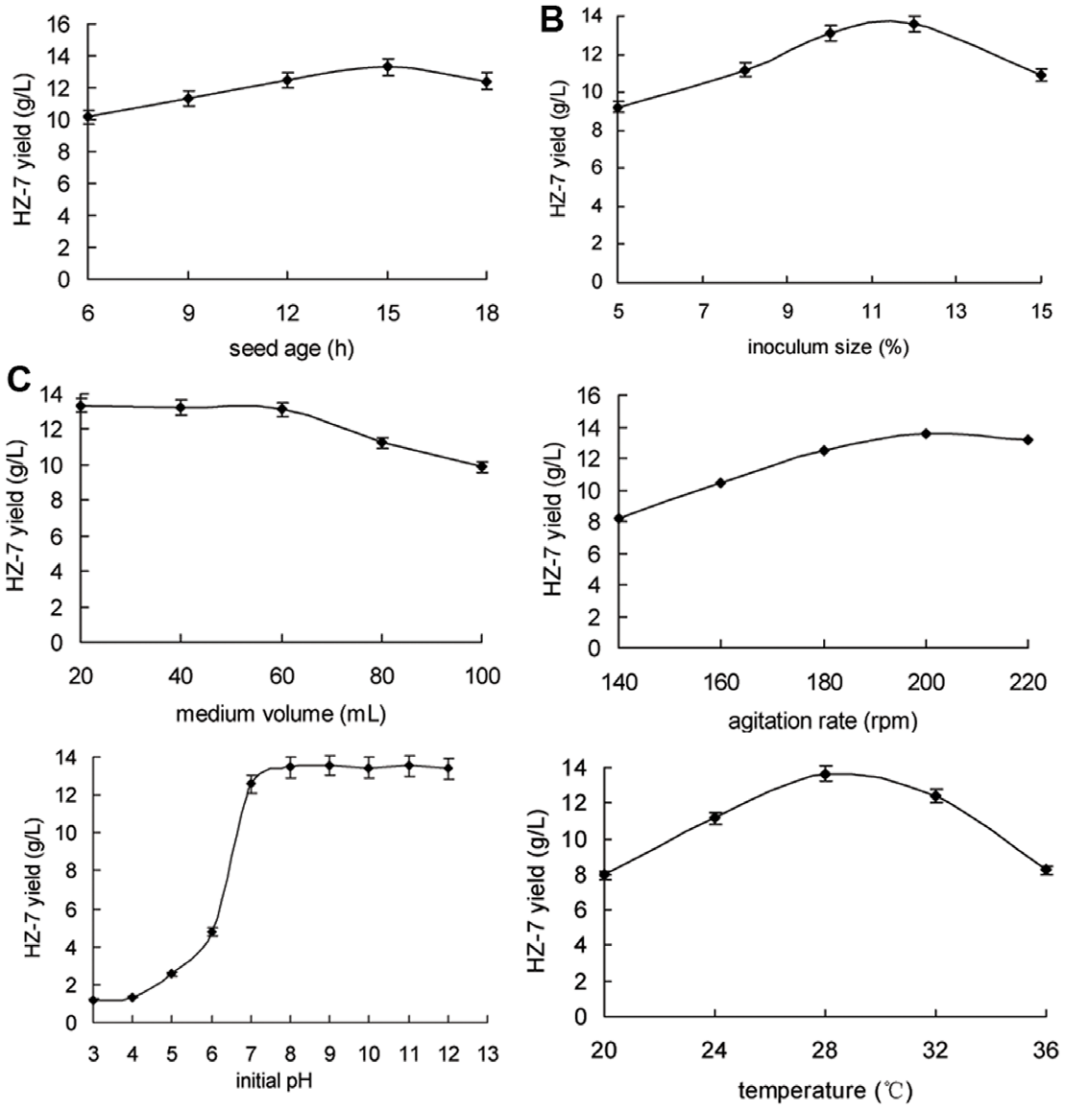
The effects of various culture constituents on HZ-7 production. A, inoculum size (%); B, seed age (h); C, medium volume (mL); D, agitation rate (rpm); E, initial pH; F, incubation temperature (°C). Each bar represented the mean± SD of three replicates.

Seed age and inoculum size were the two crucial biological parameters to be optimized. These experiment parameters generally affected the length of the lag phase. In order to a healthy inoculum, different seed ages (6, 9, 12, 15 and 18 h) were used. Maximum HZ-7 (13.31 g/L) was obtained with 15-h-old inoculum (Figure 4A). Similarly the fermentation medium was inoculated with different inoculum sizes of 5, 8, 10, 12 and 15% v/v and the results are shown in Figure 4B. Maximum yield of HZ-7 (13.63 g/L) was achieved with 12% (v/v) of 15 h old inoculum. General studies have been carried out using 10% (v/v) 24-h-old inoculums [27], [28].

Medium volume and agitation rate were vital in order to increase aeration during fermentation process, which had a great effect on the cell growth and EPS production. Various medium volumes (20, 40, 60, 80 and 120 mL) in 250-mL flasks along with different agitation rates (140, 160, 180, 200 and 220 rpm) were used. The HZ-7 production was slightly affected under a medium volume range of 20 to 60 mL and then it declined sharply when medium volumes were more than over 60 mL (Figure 4C). In order to test the economic efficiency of the fermentation processes, 50 mL of medium in a 250-mL flask was selected for HZ-7 production. Furthermore, the yield of HZ-7 was significantly increased with an agitation rate in the range of 140 to 200 rpm (Figure 4D).

Initial pH and incubation temperature were often the most influential factors during the biosynthetic process of microbial polysaccharides. Various initial pHs (3.0–12.0) and temperatures (20, 24, 28, 30, 32 and 36°C) were investigated. As shown in Figure 4E, an initial alkaline pH range among 7.0–12.0 was more favorable for HZ-7 production than acid pH values, and the maximum yield ofHZ-7 (>13.4 g/L) was obtained at the initial pH between 8.0–12.0. Figure 4F showed that the optimal temperature for HZ-7 production was 30°C corresponding to the maximum yield of 13.64 g/L.

According to above results, inoculum size, seed age and incubation temperature were three crucial factors to HZ-7 production, which were selected for BBD. The experimental design and results of the BBD were shown in Table 3. The second-order ploynomial equation was as follows:

**Table 3:** The matrix of the BBD experiment for culture condition optimization and the corresponding experimental data.

Run	*X*_1_ (temperature)		*X*_2_ (seed age)		*X*_3_ (inoculum size)		HZ-7 yeild (g/L)
	Coded level	Real level (°C)	Coded level	Real level (h)	Coded level	Real level (%)	
1	0	28	1	16	−1	8	10.45±0.06
2	1	32	−1	8	0	10	11.29±0.05
3	0	28	1	16	1	12	11.57±0.03
4	1	32	0	12	−1	8	13.34±0.06
5	−1	24	0	12	1	12	10.72±0.08
6	1	32	0	12	1	12	14.15±0.09
7	1	32	1	16	0	10	10.79±0.04
8	0	28	−1	8	−1	8	13.46±0.06
9	−1	24	0	12	−1	8	11.27±0.04
10	−1	24	−1	8	0	10	10.84±0.05
11	0	28	−1	8	1	16	13.21±0.07
12	0	28	0	12	0	12	6.78±0.04
13	−1	24	1	16	0	12	15.14±0.07
14	−1	24	0	12	1	16	15.23±0.08
15	0	28	0	12	0	12	15.31±0.06



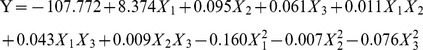
(4)


Where *Y* is HZ-7 production (g/L); −107.772 is the intercept; 8.374, 0.095 and 0.061 are the linear coefficient; 0.011, 0.043 and 0.009 are the interactive coefficients, −0.160, −0.007 and 0.076 are the quadratic coefficients; and *X*_1_, *X*_2_ and *X*_3_ are temperature, seed age and inoculum size, respectively.

**Figure 5:**
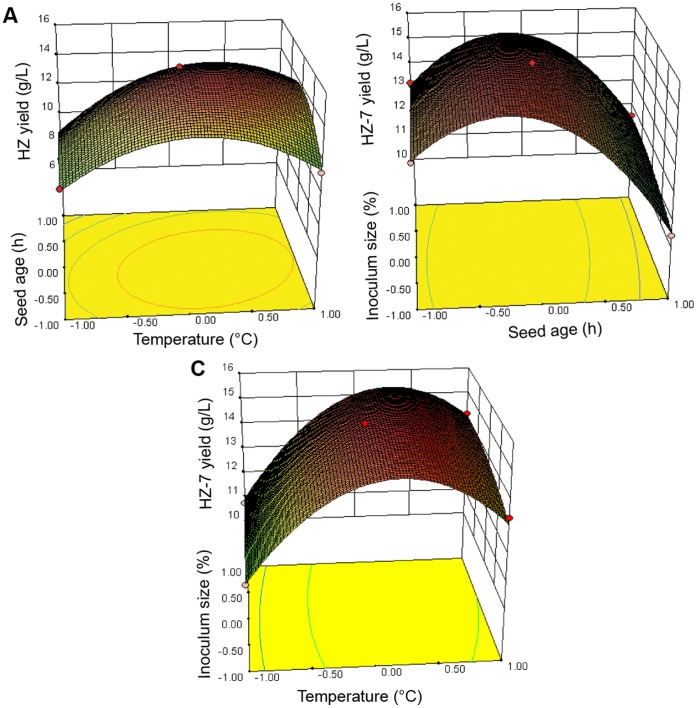
Response surface plots of the effect of three variables for culture conditions on HZ-7 production. A, interaction of temperature (*X*_1_, °C) and seed age (*X*_2_, h); B, interaction of temperature (*X*_1_, °C) and inoculum size (*X*_3_, %); C, interaction of seed age (*X*_2_, h) and inoculum size (*X*_3_, %).

The fit of the model was verified by the determination of *R* ^2^, which was 0.9964 for HZ-7 production, indicating that 99% of the variability in the response could be explained by this model. As shown in Table 4, the ‘Model *F*-value’ of 212.84 and the CV of 1.62% indicated that the model was significant. Due to less than 0.05 of *P* values, X_1_ and X_2_ were significant model terms. Moreover, the interactions between each pair of the three variables were significant, which was well supported by the fact that their strong interaction increased HZ-7 production by about 15 g/L (Figure 5A, B, C). Based on the canonical analysis, the optimal conditions of inoculum size, seed age and incubation temperature were 10.6%, 13 h and 28.9°C, respectively. The maximum yield was estimated to be 15.04 g/L, and the actual yield under the optimal fermentation conditions was 15.05 g/L, which was the average value of three experiments.

**Table 4:** Variance analysis (ANOVA) of response surface quadratic model for culture conditions of HZ-7 production.

Source	Sum ofSquares	df	MeanSquare	*F* Value	*p*-value Prob>*F*
Model	75.55	9	8.39	212.84	<0.0001^a^
*X*_1_	12.40	1	12.40	314.42	<0.0001^a^
*X*_2_	10.60	1	10.60	268.85	<0.0001^a^
*X*_3_	0.16	1	0.16	4.05	0.1004
*X*_1_ *X*_2_	3.17	1	3.17	80.34	0.0003^a^
*X*_1_ *X*_3_	0.46	1	0.46	11.72	0.0188^a^
*X*_2_ *X*_3_	0.47	1	0.47	11.90	0.0183^a^
*X*_1_^2^	24.05	1	24.05	609.77	<0.0001^a^
*X*_2_^2^	27.91	1	27.91	707.80	<0.0001^a^
*X*_3_^2^	0.34	1	0.34	8.69	<0.0320^a^
Residual	0.20	5	0.039		
Lack of Fit	0.18	3	0.061	8.42	0.1080
Pure Error	0.014	2	0.007		
Cor Total	75.74	14			

*R* ^2^ = 0.9974 Adj *R* ^2^ = 0.9927; CV = 1.62%.

a Model terms are significant.

### Properties

The main fraction of HZ-7 (HZ-7L) was obtained and used for the property investigation of HZ-7. The average molecular weight was 1.94×10^5 ^Da, which was contained in the ranges of molecular weight from other bacterial EPSs (between 5×10^3 ^Da and 50×10^6 ^Da) [5]. However this was much lower than those reported molecular weights of other *Klebsiella* sp. bacteria. (>2.0×10^6^ Da) [14], [15]. Low molecular weight may increase its water solubility and decrease its viscosity, which could be beneficial for its fermentation production and application.

Element analysis of HZ-7L showed it was composed of 39.83% C, 50.41% O and no N. Further analysis of Chemical composition revealed the presence of 91.49% total sugar and 41.67% uronic acid without any amino sugars at detected level. This higher content of uronic acid provided a certain amount of carboxyl groups to HZ-7 exopolysaccharide. The carboxyl groups presented on the molecular chain could provide more effective sites for particle attachment, thus many of the particles could easily be adsorbed to the long molecular chains, consequently working as functional moieties to generate new or modified polymer variants using different approaches.

**Figure 6:**
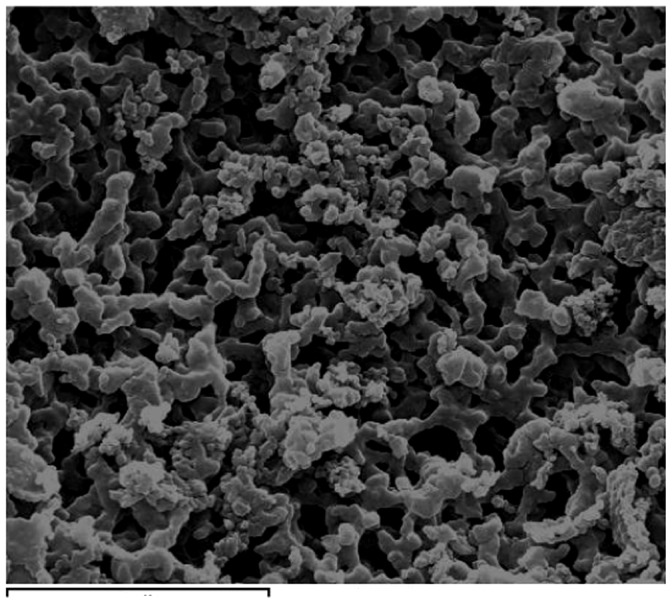
Scanning electron microphotograph of HZ-7L.

The infrared spectrum of HZ-7L displayed a broad and intense stretching peak at 3425 cm^−1^, characteristic of hydroxyl groups, C–H stretching band at 2920 cm^−1^, an asymmetrical stretching band at 1724 cm^−1^, and a symmetrical stretching band at 1616 cm^−1^ was characteristic of C = O stretching vibration. The absorption peak at 1053 cm^−1^ is characteristic for all sugar derivatives. Since the specialization of absorption peak at 1650 cm^−1^ was not seen, we can conclude that there is no amino sugar, which is in accordance with the element analysis for N and chemical detection for amino sugar. The scanning electron microscopy observations (Figure 6) indicate that the surface morphology of HZ-7L is a porous structure with randomly distributed small pores and interconnected channels, which makes its specific surface area larger and more beneficial for biosorption.

### Application of HZ-7 in Adsorption of Cr(VI)

**Figure 7:**
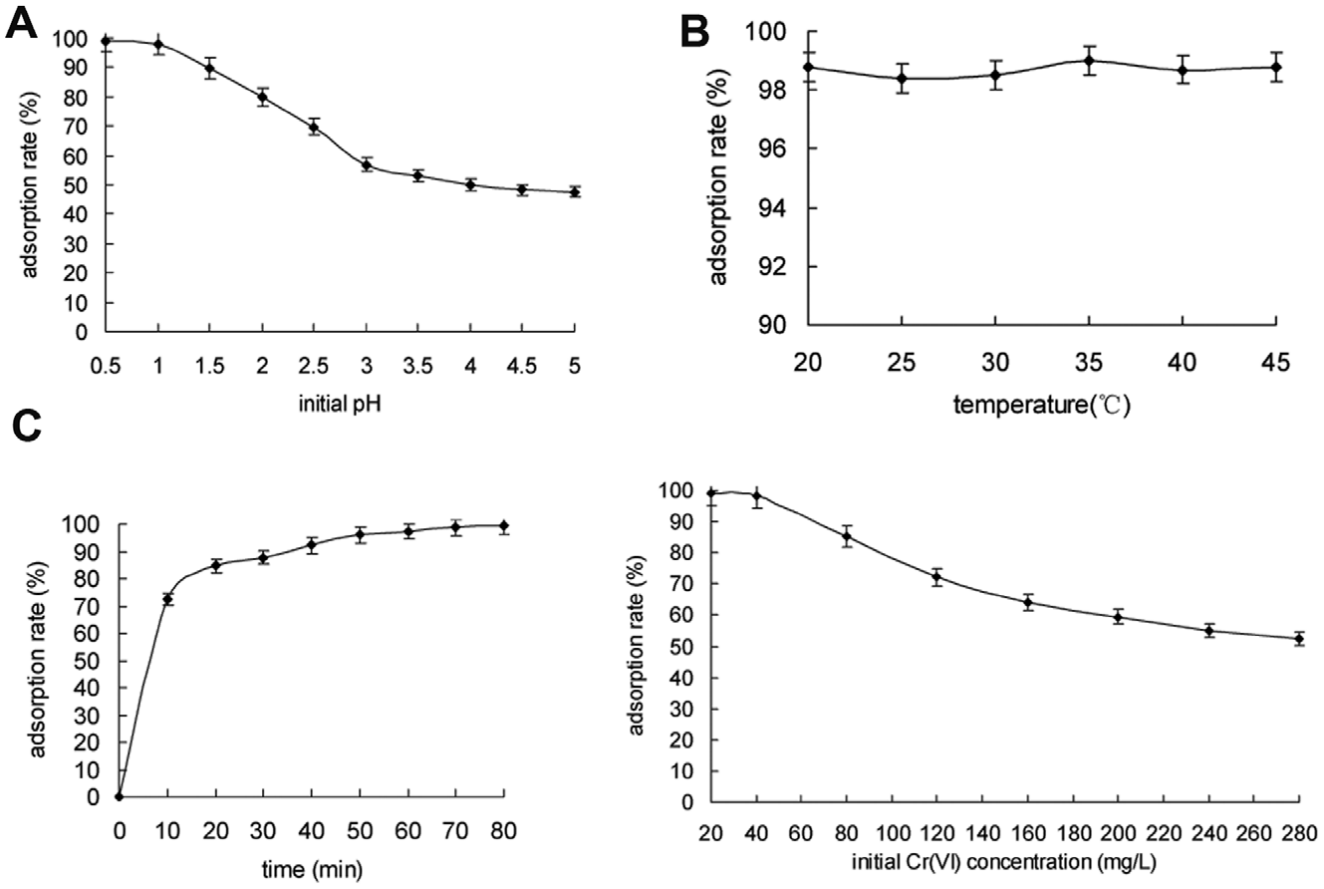
The effects of various parameters on Cr(VI) adsorption. A, initial pH; B, temperature (°C); C, adsorption time (min); D, initial concentration of Cr(VI) (mg/L). Each bar represented the mean± SD of three replicates.

The effect of the initial pH value on Cr(VI) adsorption was investigated by adjusting the solution pH to 0.5, 1.0, 1.5, 2.0, 2,5 and 3.0 with 1 M HCl. The results shown in Figure 7A demonstrated a strong adsorption under acid conditions, and the adsorption efficiency of Cr(VI) reached its maximum value (99.2%) at pH 1.0. The low constant adsorption efficiency (about 50%) was achieved at higher pH. High adsorption efficiency observed at lower pH may be attributed due to electrostatic interactions between adsorbent and adsorbate. More specifically, the increase in H^+^ concentration left the HZ-7 surface with less negative charges, meanwhile, the dominating Cr(VI) form was the negatively charged Cr_2_O_7_^−^ [29].

The effect of some ordinary ions (Na^+^, Ca^2+^, Mg^2+^ and Al^3+^) typically present in water were investigated. The results showed that no obvious interaction with the adsorption of Cr(VI). This result was similar to Chitosan adsorption [30].

To assess the effect of different temperatures, the adsorption experiments were performed at 20, 25, 30, 35 and 40°C. Surprisingly the results demonstrated that Cr(VI) adsorption was kept consistent even with change in temperature (Figure. 7B), which was different to other adsorbents [31].

The effect of contact time was presented in Figure 7C. The results showed that the adsorption of Cr(VI) increased rapidly within the first 10 min (72.5%); then the adsorption efficiency gradually increased, which attained equilibrium at about 80 min with 99% of Cr(VI) adsorption. In addition, the residual Cr(VI) in solution at equilibrium was under detected level. Cr(VI) was detected by means of titration using ammonium hydroxide, indicating no appearance of grayish-green precipitation.

The effect of initial Cr(VI) concentration (20, 40, 60, 80, 100, 120, 140, 160, 180, 200, 220, 240, 260, 280 mg/mL) was investigated in the range 20 to 280 mg/L. As shown in Figure. 7D, the Cr(VI) adsorption efficiency was 99.2% when the initial Cr(VI) concentration was 20 mg/L, however, it was reduced to be 52.4% when Cr(VI) concentration was increased to be 280 mg/L. This result indicate that HZ-7 was advantageous to absorb low concentration of Cr(VI).

**Figure 8:**
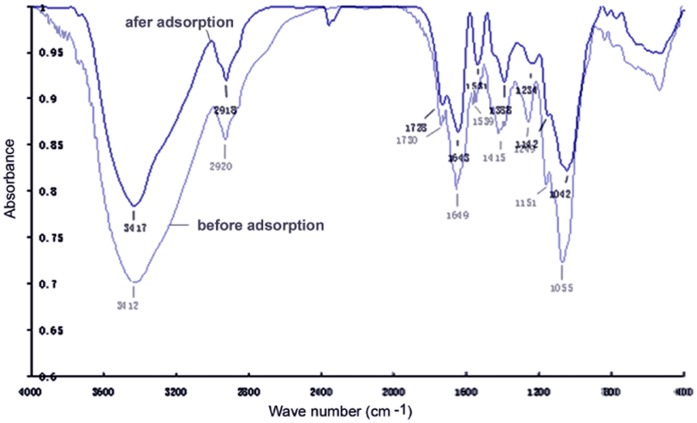
The infrared spectra of HZ-7 before and after Cr(VI) adsorption.

FTIR was employed for functional groups analysis. The FTIR spectra of HZ-7 before and after Cr(VI) adsorption are shown in Figure 8. The peak intensity of the carbonyl groups (C = O) was decreased and shifted from 3412 to 3417 cm^−1^ after Cr(VI) adsorption. In addition, the absorbance peak of the acyl groups (C–O) was shifted from 1643 to 1649 cm^−1^. These results suggest that the carbonyl groups and acyl groups might be involved in the mechanism of chromium adsorption.
